# Activation of dorsal horn cannabinoid CB2 receptor suppresses the expression of P2Y_12_ and P2Y_13_ receptors in neuropathic pain rats

**DOI:** 10.1186/s12974-017-0960-0

**Published:** 2017-09-12

**Authors:** Juan Niu, Dujuan Huang, Rui Zhou, MingXia Yue, Tao Xu, Junna Yang, Li He, Hong Tian, XiaoHong Liu, Junwei Zeng

**Affiliations:** Department of Physiology, Zunyi Medical College, Zunyi, Guizhou province 563006 China

**Keywords:** CB2 receptor, P2Y_12_ receptor, P2Y_13_ receptor, Dorsal spinal cord, Microglia, Neuropathic pain

## Abstract

**Background:**

More evidence suggests that dorsal spinal cord microglia is an important site contributing to CB2 receptor-mediated analgesia. The upregulation of P2Y_12_ and P2Y_13_ purinoceptors in spinal dorsal horn microglia is involved in the development of pain behavior caused by peripheral nerve injury. However, it is not known whether the expression of P2Y_12_ and P2Y_13_ receptors at spinal dorsal horn will be influenced after CB2 receptor activation in neuropathic pain rats.

**Methods:**

Chronic constriction injury (CCI) and intrathecal ADPbetaS injection were performed in rats to induce neuropathic pain. The paw withdrawal latency (PWL) was used to evaluate thermal hyperalgesia in neuropathic rats. The expression of P2Y_12_ and P2Y_13_ receptors, p-p38MAPK, and NF-kappaBp65 was detected with RT-PCR and western blotting analysis.

**Results:**

Treatment with AM1241 produces a pronounced inhibition of CCI-induced thermal hyperalgesia and significantly inhibited the increased expression of P2Y_12_ and P2Y_13_ receptors at the mRNA and protein levels, which open up the possibility that P2Y_12_ and P2Y_13_ receptor expression are downregulated by CB2 receptor agonist AM1241 in CCI rats. Western blot analysis demonstrated that AM1241 reduced the elevated expression of p-p38MAPK and NF-κBp65 in the dorsal spinal cord induced by CCI. After administration with either SB203580 (p38MAPK inhibitor) or PDTC (NF-kappaB inhibitor), the levels of P2Y_13_ receptor expression in the dorsal spinal cord were lower than those in the CCI group. However, in CCI rats, the increased expression of P2Y_12_ receptor was prevented by intrathecal administration of PDTC but not by SB203580. In addition, minocycline significantly decreased the increased expression of P2Y_12_ and P2Y_13_ receptors. The similar results can be observed in ADPbetaS-treated rats. Intrathecal injection of ADPbataS causes thermal hyperalgesia and increased expression of P2Y_12_ and P2Y_13_ receptors in the dorsal spinal cord of naive rats. Moreover, intrathecal injection of AM1241 alleviates pain response and reduces the elevated expression of P2Y_12_ and P2Y_13_ receptors, p-p38MAPK, and NF-κBp65 in the dorsal spinal cord of ADPbetaS-treated rats. Intrathecal injection of SB203580 significantly inhibited the ADPbetaS-induced P2Y_13_ receptor expression, without affecting P2Y_12_ receptor expression. However, treatment with either SB203580 or PDTC effectively inhibited P2Y_13_ receptor expression compared to ADPbetaS-treated rats.

**Conclusions:**

In CCI- and ADPbetaS-treated rats, AM1241 pretreatment could efficiently activate CB2 receptor, while inhibiting p38MAPK and NF-kappaB activation in the dorsal spinal cord. CB2 receptor stimulation decreased P2Y_13_ receptor expression via p38MAPK/NF-kappaB signaling. On the other hand, CB2 receptor activation decreased P2Y_12_ receptor expression via p38MAPK-independent NF-kappaB signaling pathway.

## Background

It is well known that the endocannabinoid system is an important neuromodulatory system that mediates a broad range of physiological processes [1, 2]. Endogenous cannabinoids and its receptors can regulate some biological processes, such as nervous system development, immune homeostasis, and the response to multiple endogenous and environmental insults. Endocannabinoids exert numerous effects via interaction with G-protein-coupled CB1 and CB2 receptors. More recent studies show that activation of cannabinoid CB receptor reduces the spontaneous pain behavior as well as long-lasting hyperalgesia and allodynia that result from noxious chemical stimuli and peripheral inflammation [3]. Even more, Anand et al. reported that activation of CB receptors either directly or indirectly enhances the analgesic effect of non-steroidal anti-inflammatory drugs (NSAIDs) [4].

Microglia cells are macrophage-like resident immune cells that participate in the development of some nervous system diseases. Neuroimmune activation of microglia contributes to the generation and maintenance of neuropathic pain after peripheral nerve injury. Recently, increasing evidence suggests that the dorsal spinal cord is an important site contributing to CB2 receptor-mediated analgesia [5, 6]. The increased CB2 receptor expression after peripheral nerve injury is highly restricted within the lumbar spinal cord microglia [6]. Moreover, the same pattern of CB2 receptor expression was observed in the model of spinal nerve transaction, osteoarthritis, and post-ischemic pain [7–9]. In addition, CB2 messenger RNA (mRNA) is also present in cultured spinal cord microglia cells [10].

There is a wealth of evidence to suggest that CB2-preferring agonist produces analgesia through a variety of mechanisms. Intrathecal treatment with CB2-preferring agonist reduces pain hypersensitivity by modulating the spinal immune response and microglia function in chronic pain condition [11, 12]. JWH-015 (CB2 receptor agonist) reduces the expression of interleukin-1beta (IL-1beta), interleukin-6 (IL-6), interleukin-18 (IL-18), and tumor necrosis factor-alpha (TNF-alpha) expression in the spinal cord, thereby displaying an analgesic effect [12]. Gu et al. reported that JWH-015 might relieve cancer pain by reducing NR2B-dependent activity in the rat dorsal spinal cord [13].

We noticed that the interaction between cannabinoid and P2 purinoceptors have been reported in previous studies. Cannabinoids can modulate the purinergic component of sympathetic neurotransmission in the perfused mesenteric vascular bed of rats [14]. ATP-induced 2-arachidonoylglycerol production is mediated through purinergic P2X_7_ receptor in cultured mouse astrocytes [15]. In addition, cannabinoid treatment attenuates the slow response to ATP mediated by P2X_2_ and P2X_2/3_ receptors in rat DRG neurons, which indicate that cannabinoids may inhibit nociceptive responses produced by P2X receptors [16]. Despite these observational findings, it is not clear whether the interaction of CB2 receptor and P2Y purinoceptors is involved in CB2 receptor-mediated analgesia.

More evidence indicates that dorsal horn microglia express various P2Y purinoceptors, and of these, P2Y_6_, P2Y_12_, and P2Y_13_ receptor subtypes might share a role in the pathogenesis of neuropathic pain that can occur after peripheral nerve injury. Kobayashi et al. demonstrated that there was a dramatic increase in P2Y_6_, P2Y_12_, P2Y_13_, and P2Y_14_ receptor expression in rat dorsal spinal cord microglia after spared nerve injury [17]. Intrathecal injection of the specific P2Y_6_ antagonist MRS2578, specific P2Y_12_ antagonist MRS2395, specific P2Y_13_ antagonist MRS2211, or P2Y_14_ antisense locked nucleic acids (AS-LNA) attenuated mechanical pain hypersensitivity [17]. We noticed that the highest degree (49%) of sequence identity is found between P2Y_12_ and P2Y_13_ receptors [18]. For this reason, in the current study, we examined whether the expression of P2Y_12_ and P2Y_13_ receptors can be influenced after CB2 receptor activation by using RT-PCR and western blot assay.

## Methods

### Animals

Adult male Sprague-Dawley rats (200–220 g) were used in the present study. All animals were housed (one rat per cage) in a standard 12-h light/dark cycle. The protocol was prepared from SD rats in accordance with the National Institutes of Health guidelines in a manner that minimized animal suffering and animal numbers. All experiments were carried out in accordance with China animal welfare legislation and were approved by the Zunyi Medical College Committee on Ethics in the Care and Use of Laboratory Animals.

### Implantation of intrathecal catheter

Lumbosacral intrathecal catheters were constructed and implanted as detailed in a previous study [19]. Under anesthesia with pentobarbital sodium (40 mg/kg, i.p.), the rats were fixed and a 2-cm longitudinal incision was made above vertebrae L_5–6_. Polyethylene catheters (PE-10) were pushed through the intervertebral space until a sudden movement of the tail or the hind limb were observed and then passed gently 2 cm upward to reach the level of the lumbar enlargement. The tip of the catheter was fixed on the neck area of the rats. Correct intrathecal placement was confirmed by injection of 2% lidocaine (10 μl) through the catheter. The catheter was judged to be intrathecal if paralysis and dragging of bilateral hind limbs occurred within 30 s of this injection. In addition, rats with signs of motor weakness were excluded from the experiment. The rats were housed individually after implantation of intrathecal catheter and allowed to recover 5 days before the chronic constriction injury (CCI) test. ADPbetaS (cat. no. A8016, Sigma) was made with 0.9% sodium chloride. Minocycline (cat. no. 13614-98-7, BBI) was made with 0.01 M PBS. AM1241 (CB2 receptor agonist, cat. no. ab120934, Abcam), SB203580 (p38 mitogen-activated protein kinase (p38MAPK) inhibitor, cat. no. ab120162, Abcam), and PDTC (nuclear factor-kappaB (NF-kappaB) inhibitor, cat. no. ab141406, Abcam) were dissolved in dimethyl sulfoxide (DMSO) and then further diluted with 0.9% sodium chloride, respectively. The medication was injected through the catheter twice per day. The presence of DMSO (< 0.1%) did not affect the thermal withdrawal latency of the rat hind paw.

### The chronic constriction injury (CCI) model

All surgical procedures were performed under strict sterile conditions. Under anesthesia with pentobarbital sodium (40 mg/kg, i.p.), the left sciatic nerve was exposed, and a 15-mm length of sciatic nerve proximal to the sciatic trifurcation was carefully dissected from the underlying tissue. Four loose ligatures (using 4.0 braided silk) were applied around the sciatic nerve at 1-mm intervals. The left sciatic nerve was only exposed but not ligated in sham-operated groups. Rats exhibiting neurologic impairments or infection were not included in this study.

### Thermal Hyperalgesia

The thermal withdrawal latency (TWL) was tested using a Plexiglas chamber over an elevated transparent glass surface. The rats (1 day before ligation, 1, 3, 5, 7, 10 and 14 days after ligation) were placed in the chamber. A radiant heat source was focused on the plantar surface of a hind paw, and a light intensity was preset to obtain a baseline latency of approximately 25 s. Each rat underwent three trials with a 5-min interval, and the mean value of the three trials was used as the TWL. A cutoff time of 30 s was used to avoid tissue damage.

### RT-PCR

The rats were terminally anesthetized with pentobarbital sodium (50 mg/kg) on days 3, 7, and 14 after CCI or sham surgery. The lumbar region of the ipsilateral spinal dorsal horn was dissected and placed into separate RNase-free 1.5 ml Eppendorf tubes at −80 °C. Total RNA was isolated by using the TRIzol (MRC Co., Cincinnati, USA) method after 24 h of PDGF-BB and Rg1 action. After purification, the RNA was eluted using RNase-free water, and its concentration and purity were estimated using Spectrophotometer (Thermo Fisher Scientific). All 260:280 absorbance ratios were in the range of 1.9–2.1. During RNA isolation, the samples were also treated with DNase (Qiagen) to remove any contaminating genomic DNA. Complementary DNA (cDNA) was subsequently synthesized from RNA using the SuperScript II reverse transcriptase kit (Invitrogen) according to the guidelines of the manufacturer. The specific primers used in this study are listed in Table 1. The final volume for qPCR was 20 μl of which 8 μl were H_2_O, 10 μl mastermix (Life Technologies), 1 μl assay-mix (Life Technologies), and 1 μl cDNA. Each qPCR was done in duplicate. Real-time PCR was performed on a LineGene Real-time PCR detection system (Bioer Technology, China). The reactions conditions were (1) 95 °C 8 min 1 cycle and (2) 95 °C 15 s and 60 °C 1 min, 40 cycles. Data were analyzed using the 2−^△△CT^ method [20].

**Table 1:** Primers used for RT-PCR

Gene	Forward reverse	Reverse
P2Y_12_ R	5′-GAAAGCACCAGATGCCAGTC-3′	5′-AGAACCTGGGTGATCTTGTAGTCTC-3′
P2Y_13_ R	5′-AACGGCATCAACCGTGAAGA-3′	5′-ATGAACTGGCATGTGTGACTGACTA-3′
β-Actin	5′-AGCCATGTACGTAGCCATCC-3′	5′-ACCCTCATAGATGGGCACAG-3′

### Western blot

The rats were terminally anesthetized with pentobarbital sodium (50 mg/kg). The lumbar region of the ipsilateral spinal dorsal horn was rapidly dissected and rinsed in cold phosphate-buffered saline then homogenized in 1 ml ice-cold chilled radioimmunoprecipitation (RIPA) lysis buffer containing 1% Nonidet P-40, 0.5% sodium deoxycholate, 0.1% sodium dodecyl sulfate, 1 μg/ml aprotinin, 100 μg/ml phenylmethylsulfonyl fluoride, 1 mM sodium orthovanadate, 1 μM batimastat (BB-94), and 1% protease inhibitor cocktail (Roche). Protein concentration was determined using a Bio-Rad Protein Assay kit (cat. no. 5000002; Bio-Rad Laboratories, Inc., Hercules, CA, USA). Total proteins were diluted in 4× loading buffer and were incubated at 100 °C for 5 min. Samples (80 μg) were then loaded onto a loading gel and separated on a Bis-Tris gel (12% gel for P2Y_12_ and P2Y_13_; 10% gel for p38MAPK; 8% gel for NF-kappaBp65). Separated proteins were transferred onto a nitrocellulose membrane at 200 mA for 2 h. Nonspecific sites were blocked for 1 h at room temperature in fat-free milk solution [10% in 0.1% Tween-Tris-buffered saline (TTBS)]. Membranes were then incubated overnight at 4 °C with the following rabbit polyclonal antibodies: anti-P2Y_12_ (cat. no. ab184411; 1:1000; Abcam, Cambridge, UK), anti-P2Y_13_ (cat. no. ab108444; 1:1000; Abcam), anti-phospho-p38 MAPK (cat. no. ab45381; 1:1000; Abcam), and anti-NF-kappaBp65 (cat. no. SAB4502611; 1:1000; Sigma). In addition, mouse monoclonal beta-actin antibody (cat. no. NB600-501; 1:1000; Novus Biologicals) was used. The secondary antibodies (goat anti-rabbit IgG: cat. no. A0208; goat anti-mouse IgG: cat. no. A0216, Beyotime Institute of Biotechnology) were diluted to 1:1000 and incubated for 1.5 h at room temperature. Subsequently, the membranes were developed using the enhanced chemiluminescence (ECL) reagent Beyo ECL plus (Beyotime Institute of Biotechnology). Images of the blots were captured using a ChemiDoc XRS system (Bio-Rad Laboratories, Inc.). The image was scanned, and band intensity was semi-quantified using Quantity One software v4.52 (Bio-Rad Laboratories, Inc.).

### Statistical analysis

The data were presented as means ± standard deviation. Statistical analysis was performed with one-way analysis of variance (ANOVA) followed by least significant difference (LSD) post hoc test when multiple comparisons were made (SPSS18.0, SPSS Inc., Chicago, IL, USA). *P* < 0.05 was considered statistically significant.

## Results

### Effects of AM1241 on hyperalgesia in CCI rats

**Fig. 1:**
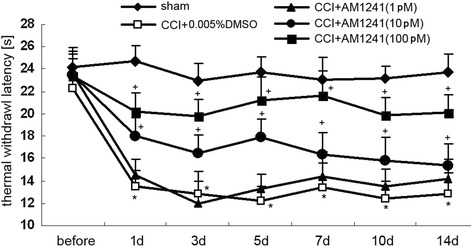
Intrathecal injection of AM1241 attenuated CCI-induced hyperalgesia. Pain sensitivity was determined by measuring TWL on the day before CCI and on days 1, 3, 5, 7, 10, and 14 after nerve injury. After nerve injury, the TWL in the vehicle-treated CCI group (0.005% DMSO) was significantly lower than that in the sham group (*P < 0.05). No significant difference was identified between the vehicle-treated CCI group and 1pM AM1241-treated CCI group. At 1, 3, 5, 7, 10, and 14 days after nerve injury, the TWL in the 10- and 100pM AM1241-treated CCI rats were significantly higher than that in the vehicle-treated CCI group (10pM: ^+^ *P* < 0.05; 100pM: ^+^ *P* < 0.05). All values represent mean ± standard deviation (*n* = 8). *P < 0.05 compared with the sham group; ^+^ *P* < 0.05 compared with the vehicle-treated CCI group

To investigate the effect of AM1241 on the thermal threshold in rats, TWL was performed on the day before (baseline) and on days 1, 3, 5, 7, 10, and 14 after CCI. Wilkerson et al. reported that AM1241 produced a dose-dependent reversal of mechanical nociceptive threshold, with maximal reversal observed at 1.5 h after AM1241 injection in CCI rats [21]. AM1241 treatment did not alter basal nociceptive thresholds to mechanical stimulation in the absence of nerve injury [22]. Then, we observed the TWL values at 1.5 h after drug administration. Figure 1 shows the time course of the antinociceptive effects produced by AM1241 at different doses. There was no significant difference in the TWL among these groups before the operation. Decreased TWL were exhibited in the CCI rats on day 1 after nerve injury compared to sham rats (*P* < 0.05), and the allodynia was sustained for 14 days. No significant difference was identified between the CCI and 1pM AM1241 treatment groups at any time points, which also means that AM1241 at 1pM had no effect on pain behaviors in the CCI rats. Compared to the vehicle-treated CCI group, AM1241 at 10pM and 100pM exerted anti-hyperalgesic effects as early as 1 day after CCI (*P* < 0.05), and the effects lasted for 14 days (10pM: *P* < 0.05; 100pM: *P* < 0.05). For this reason, we used 100pM ADPβS in all subsequent experiments.

### Effects of AM1241 on the expression of the P2Y_12_ and P2Y_13_ receptor mRNA and protein in the dorsal spinal cord of CCI rats

**Fig. 2:**
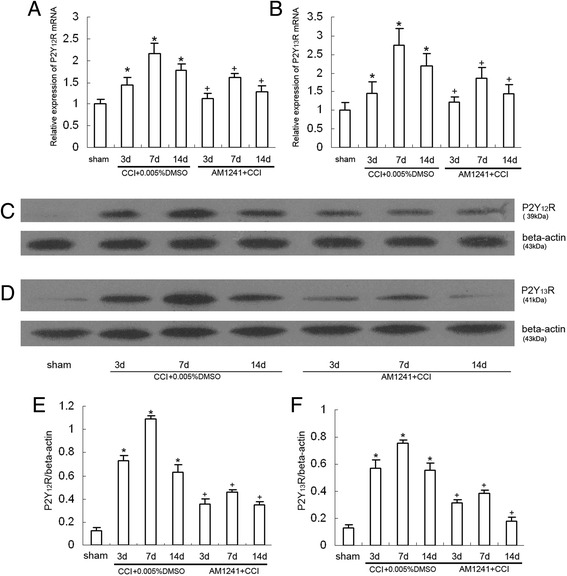
Intrathecal injection of AM1241 (100pM) attenuated CCI-induced hyperalgesia and increased expression of P2Y_12_ and P2Y_13_R in the rat dorsal spinal cord. **a** RT-PCR quantitative analysis of the P2Y_12_R mRNA expression. *P < 0.05 means comparison with the sham group; ^+^ *P* < 0.05 means comparison with the vehicle-treated CCI group. *n* = 8 per group. **b** RT-PCR quantitative analysis of the P2Y_13_R mRNA expression. *P < 0.05 means comparison with the sham group; ^+^ *P* < 0.05 means comparison with the vehicle-treated CCI group. *n* = 8 per group. **c** Western blotting image of P2Y_12_R expression. The top panel was the target band, P2Y_12_R protein, and the bottom one was for the loading control beta-actin. **d** Western blotting image of P2Y_13_R expression. The top panel was the target band, P2Y_13_R protein, and the bottom one was for the loading control beta-actin. **e** Western blotting quantitative analysis of the P2Y_12_R expression. *P < 0.05 means comparison with the sham group; ^+^ *P* < 0.05 means comparison with the vehicle-treated CCI group. *n* = 8 per group. **f** Western blotting quantitative analysis of the P2Y_13_R expression. *P < 0.05 means comparison with the sham group; ^+^ *P* < 0.05 means comparison with the vehicle-treated CCI group. *n* = 8 per group

**Fig. 3:**
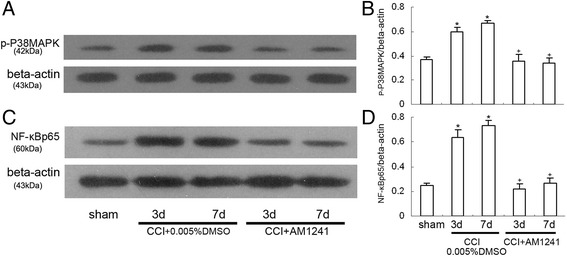
Intrathecal injection of AM1241 (100pM) attenuated CCI-induced hyperalgesia and increased expression of p-p38MAPK and NF-kappaBp65 in the rat dorsal spinal cord (*n* = 8 per group). **a** Western blotting image of p-p38MAPK expression. The top panel was the target band, p-p38MAPK, and the bottom one was for the loading control beta-actin. **b** Western blotting quantitative analysis of the p-p38MAPK expression in the rat dorsal spinal cord. *P < 0.05 means comparison with the sham group; ^+^ *P* < 0.05 means comparison with the vehicle-treated CCI group. **c** Western blotting image of NF-kappaBp65 expression. The top panel was the target band, NF-kappaBp65, and the bottom one was for the loading control beta-actin. **d** Western blotting quantitative analysis of the NF-kappaBp65 expression in the dorsal spinal cord of the rats. *P < 0.05 means comparison with the sham group; ^+^ *P* < 0.05 means comparison with the vehicle-treated CCI group

It is known that P2Y_12_ and P2Y_13_ receptors are activated in dorsal spinal horn microglia after spared nerve injury and contribute to the development and maintenance of neuropathic pain [17]. To explore whether P2Y_12_ and P2Y_13_ receptors are involved in the CB2 receptor-mediated analgesia, both mRNA and protein content of P2Y_12_ and P2Y_13_ receptors were measured by using RT-PCR and western blot. The CCI rats presented increased expression of P2Y_12_ and P2Y_13_ receptor mRNA in the ipsilateral dorsal spinal cord than the sham group (Fig. 2a, b; 3, 7, and 14 days: *P* < 0.05). After treatment with 100pM AM1241, P2Y_12_ and P2Y_13_ receptor mRNA expression were significantly lower than those in the vehicle-treated CCI group (Fig. 2a, b; 3, 7, and 14 days: *P* < 0.05). The western blot data further confirmed the results. Compared with the sham rats, P2Y_12_ and P2Y_13_ receptor expression were sharp increased to reach the peak at 7 days after CCI (Fig. 2c–f; 3, 7, and 14 days: *P* < 0.05). AM1241 (100pM) suppressed the elevated expression of P2Y_12_ and P2Y_13_ receptors (Fig. 2c–f; 3, 7, and 14 days: *P* < 0.05), which confirmed that the decreased expression of P2Y_12_ and P2Y_13_ receptors is probably mediated by CB2 receptor activation.

### The changes of p-p38MAPK and NF-kappaB expression in the dorsal spinal cord after AM1241 administration in CCI rats

p38MAPK and NF-kappaB signaling pathways within the spinal cord were considered to facilitate the perception of neuropathic pain after nerve injury [23, 24]. In particular, peripheral nerve injury causes an increase in p38MAPK phosphorylation and NF-kappaBp65 activation that also paralleled with the increased expression of some proinflammatory cytokines [25]. Then, to evaluate whether AM1241-induced antinociception is associated with p38MAPK phosphorylation and NF-kappaBp65 activation, we observed the expression of p-p38MAPK and NF-kappaBp65 in the rat dorsal spinal cord following the intrathecal injection of AM1241 (100pM) in the CCI rats. As shown in Fig. 3, compared with the sham group, the expression of p-p38MAPK and NF-kappaBp65 in the CCI rats was significantly increased at 3 and 7 days after nerve injury (3 days: *P* < 0.05; 7 days: *P* < 0.05). AM1241 (100pM) treatment significantly reduced the expression of p-p38MAPK and NF-kappaBp65 in the rat dorsal spinal cord (3 days: *P* < 0.05; 7 days: *P* < 0.05) compared with that in the vehicle-treated CCI group. It seems that AM1241 attenuates neuropathic pain through inhibiting p-p38MAPK and NF-kappaBp65 in a rat model of chronic constriction injury.

### The changes of P2Y_12_ and P2Y_13_ receptor expression in the dorsal spinal cord after SB203580 or PDTC administration in CCI rats

**Fig. 4:**
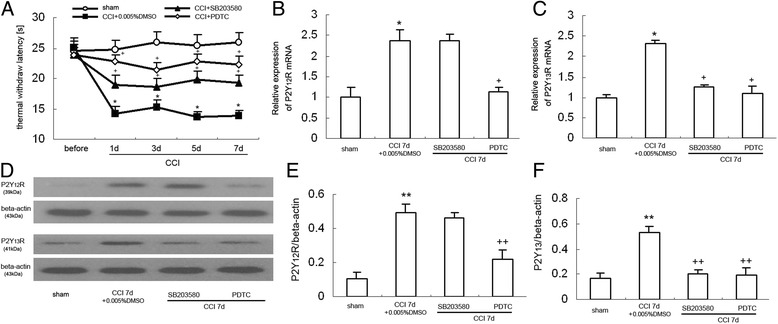
Intrathecal injection of SB203580 (50 μM) or PDTC (5 μg/10 ml) suppressed the increased expression of P2Y_12_ and P2Y_13_R in the dorsal spinal cord of CCI rats. **a** Comparison of thermal stimulation pain threshold in the ipsilateral hind paw of the rats (*n* = 8). Data are presented as TWL. CCI reduced TWL from days 1 to 7 after nerve injury. *P < 0.05 compared with control group. Pretreated with SB203580 (50 μM) or PDTC (5 μg/10 ml) significantly inhibited the reduced withdrawal threshold days 1 to 7 in CCI rats, respectively (compared with vehicle-treated CCI group, SB203580: ^+^ *P* < 0.05; PDTC: ^+^ *P* < 0.05). **b** RT-PCR results show the expression of P2Y_12_ mRNA expression in the rat dorsal spinal cord (*n* = 8 per group). *P < 0.05 means comparison with the control group; ^+^ *P* < 0.05 means comparison with the vehicle-treated CCI group. **c** RT-PCR results show the expression of P2Y_13_ mRNA expression in the rat dorsal spinal cord (*n* = 8 per group). *P < 0.05 means comparison with the control group; ^+^ *P* < 0.05 means comparison with the vehicle-treated CCI group. **d** Western blotting image of P2Y_12_R and P2Y_13_R expression. The top panel was the target band, and the bottom one was for the loading control beta-actin. **e** Western blotting quantitative analysis of the P2Y_12_R expression in the rat dorsal spinal cord. *P < 0.05 means comparison with the sham group; ^+^ *P* < 0.05 means comparison with the vehicle-treated CCI group. **f** Western blotting quantitative analysis of the P2Y_13_R expression in the rat dorsal spinal cord. *P < 0.05 means comparison with the sham group; ^+^ *P* < 0.05 means comparison with the vehicle-treated CCI group

**Fig. 5:**
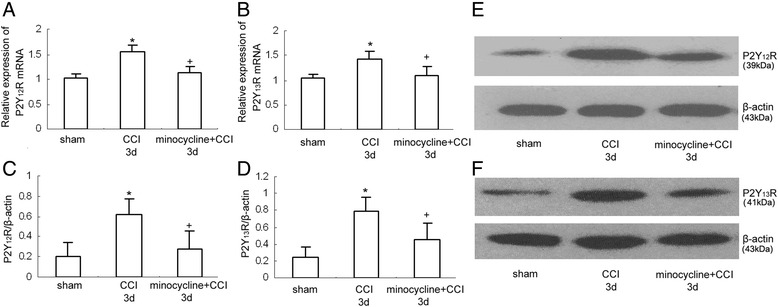
The effect of minocycline on the expression of P2Y_12_ and P2Y_13_ receptors. All values represent mean ± standard deviation. **a** RT-PCR results show the expression of P2Y_12_ mRNA expression in the rat dorsal spinal cord (*n* = 8 per group). *P < 0.05 means comparison with the sham group; ^+^ *P* < 0.05 means comparison with the CCI rats. **b** RT-PCR results show the expression of P2Y_13_ mRNA expression in the rat dorsal spinal cord (*n* = 8 per group). *P < 0.05 means comparison with the sham group; ^+^ *P* < 0.05 means comparison with the CCI rats. **c** Western blotting quantitative analysis of the P2Y_12_R expression in the rat dorsal spinal cord. *P < 0.05 means comparison with the control; ^+^ *P* < 0.05 means comparison with the ADPbetaS-treated rats. **d** Western blotting quantitative analysis of the P2Y_13_R expression in the rat dorsal spinal cord. *P < 0.05 means comparison with the control; ^+^ *P* < 0.05 means comparison with the ADPbetaS-treated rats. **e** Western blotting image of P2Y_12_R expression. The top panel was the target band, P2Y_12_R, and the bottom one was for the loading control beta-actin. **f** Western blotting image of P2Y_13_R expression. The top panel was the target band, P2Y_13_R, and the bottom one was for the loading control beta-actin

Through the analysis of these data generated in the previous experiments, we speculate that the inhibition of p38MAPK/NF-kappaB pathway is an important mechanism in CB2 receptor-mediated decreased expression of P2Y_12_ and P2Y_13_ receptors in CCI rats. To further ascertain the role of p38MAPK/NF-kappaB pathway in P2Y_12_ and P2Y_13_ receptor expression in rats after nerve ligation, the p38MAPK inhibitor SB203580 and NF-kappaB inhibitor PDTC were applied. We observed the TWL values at 1.5 h after drug injection in every group. SB203580 (50 μmol/l) and PDTC (5 μg/10 ml) treatments did not alter basal nociceptive thresholds in the absence of nerve injury. The CCI group displayed significantly decreased thermal withdrawal latency on days 1, 3, 5, and 7 compared with the sham rats (Fig. 4a: *P* < 0.05), which were markedly increased by SB203580 (Fig. 4a: *P* < 0.05) or PDTC (Fig. 4a: *P* < 0.05), respectively. In Fig. 5b, c, compared with that in the vehicle-treated CCI group, SB203580 (50 μmol/l) treatment significantly suppressed the increased expression of P2Y_13_ receptor mRNA after nerve injury (3 days: *P* < 0.05; 7 days: *P* < 0.05) without affecting the expression of P2Y_12_ receptor mRNA. However, compared with that in the vehicle-treated CCI group, SB203580 (50 μmol/l) or PDTC (5 μg/10 ml) treatment significantly suppressed the increased expression of P2Y_13_ receptor after nerve injury (SB203580: 3 days: *P* < 0.05; 7 days: *P* < 0.05; PDTC: 3 days: *P* < 0.05; 7 days: *P* < 0.05). It seems that p38MAPK/NF-kappaB pathway may play an important role in CCI-induced increased expression of P2Y_13_ receptor. However, the increased expression of P2Y_12_ receptor may be regulated via p38MAPK-independent NF-kappaB pathway.

### The changes of P2Y_12_ and P2Y_13_ receptor expression in the dorsal spinal cord of after minocycline administration in CCI rats

Mounting studies indicate that spinal microglia activation often promotes the activation of a series of genes and neurotransmitters, which leads to chemokine secretion and pain hypersensitivities. In different models of neuropathic pain, the phosphor-p38MAPK in the rat dorsal spinal cord was found exclusively in microglia, but not in neurons or astrocytes [23, 26, 27]. In addition, Yin et al. reported that the NF-κB-IR cells represented neurons, microglia, and astrocytic cells in the spinal cord on day 7 after CCI surgery [28]. In order to identify whether reduction of microglia activity can influence the expression of P2Y_12_ and P2Y_13_ receptors, we observed the effect of intrathecal administration of minocycline (5μg/10μ﻿l﻿﻿) on the expression of these two receptors in the CCI rats. In our present experiments, results from RT-PCR and western blot support the idea that dorsal spinal cord P2Y_12_ and P2Y_13_ receptors play an important role in the maintenance of neuropathic pain. Moreover, compared with that in the CCI rats, intrathecal administration of minocycline resulted in lower expression of P2Y_12_ and P2Y_13_ receptors at mRNA or protein level (mRNA: *P* < 0.05; protein: *P* < 0.05)*.* It seems that minocycline could efficiently decrease microglia activity, which results in downregulation of P2Y_12_ and P2Y_13_ receptor expression.

### The changes of P2Y_12_ and P2Y_13_ receptor expression in the dorsal spinal cord of ADPβS-treated rats

**Fig. 6:**
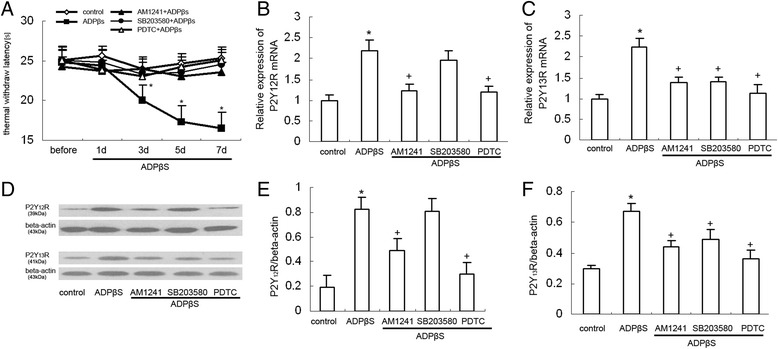
The effect of AM1241, SB203580, and PDTC on the thermal threshold and the expression of P2Y_12_ and P2Y_13_ receptors. Data are presented as TWL. All values represent mean ± standard deviation. **a** Comparison of thermal stimulation pain threshold in the ipsilateral hind paw of rats (*n* = 8). Spinal ADPbetaS injection reduced TWL from days 3 to 7 after ADPbetaS injection. *P < 0.05 compared with control group. Pretreated with AM1241 (100 PM, 20 min), SB203580 (50 μM), or PDTC (5 μg/10 μl) significantly inhibited the reduced withdrawal threshold days 3 to 7 in ADPbetaS-treated rats, respectively (compared with ADPbetaS-treated rats, AM1241: *P* < 0.05; SB203580: *P* < 0.05; PDTC: *P* < 0.05). **b** RT-PCR results show the expression of P2Y_12_ mRNA expression in the dorsal spinal cord of the rats in every groups (*n* = 8 per group). *P < 0.05 means comparison with the control group; ^+^ *P* < 0.05 means comparison with the ADPbetaS-treated rats. **c** RT-PCR results show the expression of P2Y_13_ mRNA expression in the dorsal spinal cord of the rats in every group (*n* = 8 per group). *P < 0.05 means comparison with the control group; ^+^ *P* < 0.05 means comparison with the ADPbetaS-treated rats. **d** Western blotting image of P2Y_12_R expression. The top panel was the target band, P2Y_12_R, and the bottom one was for the loading control beta-actin. **e** Western blotting quantitative analysis of the P2Y_12_R expression in the dorsal spinal cord of the rats. *P < 0.05 means comparison with the control; ^+^ *P* < 0.05 means comparison with the ADPbetaS-treated rats. **f** Western blotting quantitative analysis of the P2Y_13_R expression in the dorsal spinal cord of the rats. *P < 0.05 means comparison with the control; ^+^ *P* < 0.05 means comparison with the ADPbetaS-treated rats

Tatsumi et al. reported that intrathecal injection of 2Me-SADP induced mechanical hypersensitivity in SD rats [29]. In the present study, we found intrathecal injection of ADPbetaS have the similar effect on naive rats. ADPbetaS, a non-selective P2Y receptor agonist, is widely used in pharmacological studies of purine and related compounds. The agonist ADPbetaS activates the G-protein-coupled purine P2Y_1_, P2Y_12_, and P2Y_13_ receptors [18]. Kobayashi et al. reported that P2Y_1_ mRNA was expressed in some dorsal horn neuron and astrocyte [30]. P2Y_12_ and P2Y_13_ receptors were rather selectively expressed in dorsal spinal cord microglia [17]. Thermal hyperalgesia was induced by repeated intrathecal injection of ADPbetaS (100 μM) for seven consecutive days. As shown in Fig. 6a, the ADPbetaS-treated rats displayed significantly decreased thermal withdrawal latency on days 3, 5, and 7 compared with the sham groups (*P* < 0.05), which were markedly increased by AM1241 (100pM, *P* < 0.05), SB203580 (50 μmol/l, *P* < 0.05), or PDTC (5 μg/10 ml, *P* < 0.01), respectively. Compared with the control group, P2Y_12_ and P2Y_13_ receptor expression at mRNA levels in ADPbetaS-treated rats was significantly increased at 7 days after ADPbetaS administration (Fig. 6b, c: *P* < 0.05). After treatment with AM1241 (100pM), P2Y_12_ and P2Y_13_ receptor expression at mRNA levels were significantly lower compared with the ADPbetaS-treated rats (Fig. 6b, c: *P* < 0.05), which largely confirmed that CB2 receptor stimulation would lead to the decreased expression of P2Y_12_ and P2Y_13_ receptors. Western blot experiments showed similar results. In ADPbetaS-treated rats, the expression of P2Y_12_ and P2Y_13_ receptors at the protein levels were obviously elevated (Fig. 6d). After treatment with AM1241 (100pM), P2Y_12_ and P2Y_13_ receptor expression at the protein levels were significantly lower compared with the CCI group (Fig. 6e, f: P2Y_12_: *P* < 0.05; P2Y_13_: *P* < 0.05).

To further explore whether p38MAPK/NF-κB pathway are necessary for the increased expression of P2Y_12_ and P2Y_13_ receptors in the ADPbetaS-treated rats, we observed the effect of intrathecal injection of SB203580 and PDTC on expression of these two receptors in ADPbetaS-treated rats. SB203580 and PDTC were injected 30 min before AM1241 administration. RT-PCR showed that both SB203580 and PDTC treatment effectively inhibited P2Y_13_ receptor expression in the dorsal spinal cord of the ADPbetaS-treated rats (Fig. 6c, SB203580: *P* < 0.05; PDTC: *P* < 0.05). Similarly, western blot analysis also demonstrated both SB203580 and PDTC treatment effectively inhibited P2Y_13_ receptor protein expression in the dorsal spinal cord of the ADPbetaS-treated rats (Fig. 6f, SB203580: *P* < 0.05; PDTC: *P* < 0.05). On the other hand, we noticed that the increased P2Y_12_ receptor expression at mRNA and protein levels can be suppressed by PDTC (Fig. 6b, e, PDTC: *P* < 0.05), but not be impaired by SB203580. These observations suggest that p38MAPK/NF-kappaB pathway may play an important role in ADPbetaS-induced increased expression of P2Y_13_ receptor. However, the increased expression of P2Y_12_ receptor may be regulated via p38MAPK-independent NF-kappaB pathway.

## Discussion

During recent years, more and more studies suggest the existence of a crosstalk between endocannabinoids and other neurotransmitter systems in the nervous system. Varodayan et al. reported that CB1 receptor activation decreased the frequency of spontaneous and miniature GABAA receptor-mediated inhibitory postsynaptic currents [31]. Cannabinoids also inhibit purinergic components of sympathetic neurotransmission in rat mesenteric bed [14]. Tatsumi et al. suggest that spared nerve injury induced P2Y_12_ and P2Y_13_ receptor activation in rat spinal dorsal horn microglia [29]. In this study, we observed the effect of CB2 receptor activation on P2Y_12_ and P2Y_13_ receptor expression in the spinal dorsal horn in neuropathic pain rats. Chronic constriction injury model of neuropathic pain shows some symptoms that are common in human neuropathic pain patients including allodynia and mechanical and thermal hyperalgesia. In this study, using this model, we found that the expression levels of the dorsal spinal cord P2Y_12_ and P2Y_13_ receptors increased. The level of mRNA and protein of P2Y_12_ and P2Y_13_ receptors was suppressed by intrathecally administered AM1241 in the CCI rats. In order to further validate the results, ADPbetaS-treated rats were used. We found that, in the ADPbetaS-treated rats, the increased expression of P2Y_12_ and P2Y_13_ receptors in the dorsal spinal cord were significantly inhibited after AM1241 administration. It seems that stimulation of CB2 receptor can repress P2Y_12_ and P2Y_13_ receptor gene transcription, thereby displaying an analgesic effect in the CCI rats.

Overwhelming evidences indicate an important role for endocannabinoid-CB2 receptor signaling in a large number of the major pathologies including cardiovascular, gastrointestinal, neurodegenerative, psychiatric, and many other diseases [14, 32]. JWH133 (CB2 receptor agonist) could efficiently attenuate paraquat-induced p38MAPK and NF-kappaB activation in lung tissue of rats with paraquat-induced acute lung injury [32]. In AβPP/PS1 mice, JWH133 reduced expression of active p38MAPK and some proinflammatory cytokines [33]. CB2 receptor, expressed in microglia, is an important pathological modulator in regulating immune responses and inflammation in the nerve system. In rat forebrain microglia, CB2 receptor agonist attenuates LPS-induced reactive oxygen species (ROS) generation partly through reduction of NF-kappaB activation [34]. In BV2 microglia, CB2 receptor activation suppresses the hypoxia-induced neuroinflammatory response through inhibition of NF-kappaB activation [35]. Immunofluorescence studies have shown that the levels of p-p38 (phosphorylated p38) are particularly increased in the spinal microglia after nerve injury but are not increased in the neurons and astrocytes [23, 26, 36]. Intrathecal administration of SB203580 suppresses development of the peripheral nerve injury-induced tactile allodynia [26, 29]. In addition, several studies have demonstrated that pNF-kappaB contribute to the transmission of nociceptive signals in inflammatory and neuropathic states [25, 26, 36–39]. Chu et al. observed that atorvastatin may primarily inhibit the nuclear translocation of pNFκB to prevent CCI-induced peripheral neuropathic pain. In addition, double immunofluorescent staining further demonstrated that pNFκB proteins were decreased by atorvastatin in DRG satellite cells and spinal microglia [37]. Moreover, Wang et al. reported that the activation of spinal cord microglial p38/NF-kappaB pathway is also involved in the development of tolerance to morphine-induced analgesia [39]. To further explore the underlying mechanisms of AM1241 alleviating neuropathic pain, we observed the effect of AM1241 on the expression of p-p38MAPK and NF*-*kappaBp65. It seems that the decreased expression of p-p38MAPK and NF*-*kappaBp65 in microglia may contribute to CB2 receptor-mediated analgesia.

In the present study, minocycline suppressed the elevated expression of P2Y_12_ and P2Y_13_ receptors in the CCI rats, which also further confirmed the role of dorsal spinal cord microglia activation in the regulation of these two purinoceptor expression. Microglia p38MAPK/NF-kappaB signaling pathway is a multi-component pathway that regulates the expression of hundreds of genes that are involved in diverse and key cellular and organismal processes. Inhibition of p38MAPK activation by SB203580 also decreased the activation of NF-kappaB and the production of proinflammatory cytokines [40, 41]. There are some similar findings which discerned that NF-kappaBp65 regulates P2X_3_ and P2Y_2_ receptor transcription [42, 43]. In the present study, we found that peripheral nerve injury induced an increased expression of p-p38MAPK and NF-kappaBp65 that paralleled with the marked increase of the expression of P2Y_13_ receptors. Furthermore, AM1241 pretreatment significantly inhibited the expression of p-p38MAPK and NF-kappaBp65, and this suppression is paralleled with the decreased P2Y_13_ receptor expression. Moreover, inhibition of p38MAPK/NF-kappaB signaling pathway decreased the expression of P2Y_13_ receptor in the CCI rats. Thus, our results raise a possibility that p38MAPK/NF-kappaB signaling pathway may be involved in CB2 receptor-mediated decreased expression of P2Y_13_ receptor because upregulation of P2Y_13_ receptor expression was markedly reduced by SB203580 and PDTC, respectively. In the ADPbetaS-treated rats, the increased expression of P2Y_13_ receptor was inhibited after SB203580 or PDTC treatment, which largely confirmed that downregulation of p38MAPK/NF-kappaB signaling is necessary for the decreased expression of P2Y_13_ receptor after CB2 receptor activation.

p38MAPK-dependent and p38MAPK-independent NF-kappaB signaling pathway was reported in some different types of cells [39, 40, 44–46]. Wang et al. reported that focal adhesion kinase activates NF-kappaB via ERK1/2 and p38MAPK pathways in Aβ(25–35)-induced apoptosis of differentiated PC12 cells [46]. NADPH oxidase/Akt/NF-kappaB, but not p38MAPK, is involved in CCL2 production in endothelial cells stimulated with LPS [47]. In the present study, the increased expression of P2Y_12_ receptor in CCI rats was not significantly altered after SB203580 pretreatment, which imply that the expression of P2Y_12_ receptor is not mediated by p38MAPK. However, the increased expression of P2Y_12_ receptor was significantly inhibited after PDTC pretreatment, which imply that downregulation of NF-kappaB signaling is necessary for the decreased expression of P2Y_12_ receptor after PDTC pretreatment. Similarly, in the ADPbetaS-treated rats, the increased expression of P2Y_12_ receptor was inhibited by PDTC but was not influenced by SB203580. It is most likely that the increased expression of P2Y_12_ receptor is probably mediated through p38MAPK-independent NF-kappaB signaling. Jin et al. also suggest that PI_3_K/Akt pathway localized in spinal microglia is involved in both neuropathic and inflammatory pain [48]. Lee et al. also discerned that PI_3_K/Akt is involved in the expression of inflammatory mediators in microglia through the activation of NF-kappaB [49]. However, whether PI_3_K/Akt/NF-kappaB is necessary for the decreased expression of P2Y_12_ receptor after AM1241 pretreatment awaits further investigation.

In the central nerve system, microglia cells are considered as the primary responders to noxious stimulation. In the spinal cord, CB2 receptor activation may inhibit the microglia p38MAPK and NF-kappaB activation to prevent CCI- and ADPbetaS-induced neuropathic pain. CB2 receptor stimulation decreased P2Y_13_ receptor expression via p38MAPK/NF-kappaB signaling. However, CB2 receptor activation decreased P2Y_12_ receptor expression via p38MAPK-independent NF-kappaB signaling pathway. It seems that decreased expression of P2Y_12_ and P2Y_13_ receptors may be considered one of the mechanisms involved in CB2 receptor-mediated analgesia.

## Conclusions

In the present study, we found that CB2 receptor activation significantly inhibited peripheral nerve injury-induced thermal hyperalgesia and the increased expression of P2Y_12_ and P2Y_13_ receptors in the rat dorsal spinal cord. The suppression of p38MAPK/ NF-kappaB signaling may play a role in CB2 receptor-mediated analgesia. CB2 receptor stimulation inhibits the expression of P2Y_13_ receptor via p38MAPK/NF-kappaB signaling. However, CB2 receptor activation inhibited the expression of P2Y_12_ receptor via p38MAPK-independent NF-kappaB signaling pathway. CB2 receptor activation which induced the decreased expression of P2Y_12_ and P2Y_13_ receptors may be important for pathophysiological events occurring within the spinal cord, for where it is implicated in the transduction of the “pain” message.

## Abbreviations

2Me-SADP: 2-(Methylthio)adenosine 5′-diphosphate trisodium salt hydrate
ADPbetaS: Adenosine 5¢-O-(2-thiodiphosphate)
Akt: Protein kinase B
AM1241: (2-Iodo-5-nitrophenyl)[1-[(1-methyl-2-piperidinyl)methyl]-1*H*–indol-3-yl] methanone
ATP: Adenosine triphosphate
CB2R: Cannabinoid receptor type 2
CCI: Chronic constriction injury
DMSO: Dimethyl sulfoxide
IL-18: Interleukin-18
IL-1β: Interleukin-1β
IL-6: Interleukin-6
JWH-015: (2-Methyl-1-propyl-1H-indol-3-yl)-1-naphthalenylmethanone
LNA: Locked nucleic acid
LPS: Lipopolysaccharides
LSD: Least significant difference
MRS2211: 2-[(2-Chloro-5-nitrophenyl)azo]-5-hydroxy-6-methyl-3-[(phosphonooxy)methyl]-4-pyridinecarboxaldehyde sodium salt
MRS2395: 2,2-Dimethyl-propionic acid 3-(2-chloro-6-methylaminopurin-9-yl)-2-(2,2-dimethyl-propionyloxymethyl)-propyl ester
MRS2578: 1,4-Di[3-(3-isothiocyanatophenyl)thioureido]butane
NF-kappaB: Nuclear factor-kappaB
NR2B: NMDA receptor 2B subunit
NSAIDs: Non-steroidal anti-inflammatory drugs
p38MAPK: p38 mitogen-activated protein kinase
PDTC: Ammonium pyrrolidinedithiocarbamate
PI3K: Phosphatidylinositol 3-kinase
RIPA: Radioimmunoprecipitation
ROS: Reactive oxygen species
RT-PCR: Real-time polymerase chain reaction
SB203580: 4-[4-(4-Fluorophenyl)-2-[4-(methylsulfinyl)phenyl]-1*H*-imidazol-5-yl] pyridine
TNF-alpha: Tumor necrosis factor-alpha
TTBS: Tween-Tris-buffered saline
TWL: Thermal withdrawal latency

## References

[1] Lisboa SF, Gomes FV, Guimaraes FS, Campos AC (2016). Microglial cells as a link between cannabinoids and the immune hypothesis of psychiatric disorders. Front Neurol.

[2] Huang WJ, Chen WW, Zhang X (2016). Endocannabinoid system: role in depression, reward and pain control. Mol Med Rep.

[3] Guindon J, Hohmann AG (2009). The endocannabinoid system and pain. CNS Neurol Disord Drug Targets.

[4] Anand P, Whiteside G, Fowler CJ, Hohmann AG (2009). Targeting CB2 receptors and the endocannabinoid system for the treatment of pain. Brain Res Rev.

[5] Lu C, Shi L, Sun B, Zhang Y, Hou B, Sun Y, Ma Z, Gu X (2017). A Single Intrathecal or Intraperitoneal Injection of CB2 Receptor Agonist Attenuates Bone CancerPain and Induces a Time-Dependent Modification of GRK2. Cell Mol Neurobiol.

[6] Curto-Reyes V, Boto T, Hidalgo A, Menéndez L, Baamonde A (2011). Antinociceptive effects induced through the stimulation of spinal cannabinoid type 2 receptors in chronically inflamed mice. Eur J Pharmacol.

[7] Xu J, Tang Y, Xie M, Bie B, Wu J, Yang H, Foss JF, Yang B, Rosenquist RW, Naguib M (2016). Activation of cannabinoid receptor 2 attenuates mechanical allodynia and neuroinflammatory responses in a chronic post-ischemic pain model of complex regional pain syndrome type I in rats. Eur J Neurosci.

[8] Burston JJ, Sagar DR, Shao P, Bai M, King E, Brailsford L, Turner JM, Hathway GJ, Bennett AJ, Walsh DA, Kendall DA, Lichtman A, Chapman V (2013). Cannabinoid CB2 receptors regulate central sensitization and pain responses associated with osteoarthritis of the knee joint. PLoS One.

[9] Romero-Sandoval A, Nutile-McMenemy N, DeLeo JA (2008). Spinal microglial and perivascular cell cannabinoid receptor type 2 activation reduces behavioral hypersensitivity without tolerance after peripheral nerve injury. Anesthesiology.

[10] Beltramo M, Bernardini N, Bertorelli R, Campanella M, Nicolussi E, Fredduzzi S, Reggiani A (2006). CB2 receptor-mediated antihyperalgesia: possible direct involvement of neural mechanisms. Eur J Neurosci.

[11] Romero-Sandoval A, Eisenach JC (2007). Spinal cannabinoid receptor type 2 activation reduces hypersensitivity and spinal cord glial activation after paw incision. Anesthesiology.

[12] Lu C, Liu Y, Sun B, Sun Y, Hou B, Zhang Y, Ma Z, Gu X (2015). Intrathecal injection of JWH-015 attenuates bone cancer pain via time-dependent modification of pro- inflammatory cytokines expression and astrocytes activity in spinal cord. Inflammation.

[13] Gu X, Mei F, Liu Y, Zhang R, Zhang J, Ma Z (2011). Intrathecal administration of the cannabinoid 2 receptor agonist JWH015 can attenuate cancer pain and decrease mRNA expression of the 2B subunit of N-methyl-D-aspartic acid. Anesth Analg.

[14] Pakdeechote P, Dunn WR, Ralevic V (2007). Cannabinoids inhibit noradrenergic and purinergic sympathetic cotransmission in the rat isolated mesenteric arterial bed. Br J Pharmacol.

[15] Walter L, Dinh T, Stella N (2004). ATP induces a rapid and pronounced increase in 2-arachidonoylglycerol production by astrocytes, a response limited by monoacylglycerol lipase. J Neurosci.

[16] Krishtal O, Lozovaya N, Fedorenko A, Savelyew I, Chizhmakov I (2006). The agonists for nociceptors are ubiquitous, but the modulators are specific: P2X receptors in the sensory neurons are modulated by cannabinoids. Pflugers Arch.

[17] Kobayashi K, Yamanaka H, Yanamoto F, Okubo M, Noguchi K (2012). Multiple P2Y subtypes in spinal microglia are involved in neuropathic pain after peripheral nerve injury. Glia.

[18] Abbracchio MP, Burnstock G, Boeynaems JM, Barnard EA, Boyer JL, Kennedy C, Knight GE, Fumagalli M, Gachet C, Jacobson KA, Weisman GA (2006). International Union of Pharmacology LVIII: update on the P2Y G protein-coupled nucleotide receptors: from molecular mechanisms and pathophysiology to therapy [J]. Pharmacol Rev.

[19] Pogatzki EM, Zahn PK, Brennan TJ (2000). Lumbar catheterization of the subarachnoid space with a 32-gauge polyurethane catheter in the rat. Eur J Pain.

[20] Livak KJ, Schmittgen TD (2001). Analysis of relative gene expression data using real-time quantitative PCR and the 2−^△△CT^ method. Methods.

[21] Wilkerson JL, Gentry KR, Dengler EC, Wallace JA, Kerwin AA, Kuhn MN, Zvonok AM, Thakur GA, Makriyannis A, Milligan ED (2012). Immunofluorescent spectral analysis reveals the intrathecal cannabinoid agonist, AM1241, produces spinal anti-inflammatory cytokine responses in neuropathic rats exhibiting relief from allodynia. Brain Behav.

[22] Gutierrez T, Crystal JD, Zvonok AM, Makriyannis A, Hohmann AG (2011). Self-medication of a cannabinoid CB2 agonist in an animal model of neuropathic pain. Pain.

[23] Choi DC, Lee JY, Lim EJ, Baik HH, Oh TH, Yune TY (2012). Inhibition of ROS-induced p38MAPK and ERK activation in microglia by acupuncture relieves neuropathicpain after spinal cord injury in rats. Exp Neurol.

[24] Xia L, Zhang Y, Dong T (2016). Inhibition of microRNA-221 alleviates neuropathic pain through targeting suppressor of cytokine signaling 1. J Mol Neurosci.

[25] Zhou CH, Li X, Zhu YZ, Huang H, Li J, Liu L, Hu Q, Ma TF, Shao Y, Wu YQ (2014). Ghrelin alleviates neuropathic pain through GHSR-1a-mediated suppression of the p38 MAPK/NF-κB pathway in a rat chronic constriction injury model. Reg Anesth Pain Med.

[26] Tsuda M, Mizokoshi A, Shigemoto-Mogami Y, Koizumi S, Inoue K (2004). Activation of p38 mitogen-activated protein kinase in spinal hyperactive microglia contributes topain hypersensitivity following peripheral nerve injury. Glia.

[27] Ding Y, Shi W, Xie G, Yu A, Wang Q, Zhang Z (2015). CX3CR1 Mediates nicotine withdrawal-induced hyperalgesia via microglial P38 MAPK signaling. Neurochem Res.

[28] Yin Q, Fan Q, Zhao Y, Cheng MY, Liu H, Li J, Lu FF, Jia JT, Cheng W, Yan CD (2015). Spinal NF-κB and chemokine ligand 5 expression during spinal glial cell activation in a neuropathicpain model. PLoS One.

[29] Tatsumi E, Yamanaka H, Kobayashi K, Yagi H, Sakagami M, Noguchi K (2015). RhoA/ROCK pathway mediates p38 MAPK activation and morphological changes downstream of P2Y12/13 receptors in spinal microglia in neuropathic pain. Glia.

[30] Kobayashi K, Fukuoka T, Yamanaka H, Dai Y, Obata K, Tokunaga A, Noguchi K (2006). Neurons and glial cells differentially express P2Y receptor mRNAs in the rat dorsal root ganglion and spinal cord. J Comp Neurol.

[31] Varodayan FP, Soni N, Bajo M, Luu G, Madamba SG, Schweitzer P, Parsons LH, Roberto M (2016). Chronic ethanol exposure decreases CB1 receptor function at GABAergic synapses in the rat central amygdala. Addict Biol.

[32] Liu Z, Wang Y, Zhao H, Zheng Q, Xiao L, Zhao M (2014). CB2 receptor activation ameliorates the proinflammatory activity in acute lung injury induced by paraquat. Biomed Res Int.

[33] Aso E, Juvés S, Maldonado R, Ferrer I (2013). CB2 cannabinoid receptor agonist ameliorates Alzheimer-like phenotype in AβPP/PS1 mice. J Alzheimers Dis.

[34] Ribeiro R, Wen J, Li S, Zhang Y (2013). Involvement of ERK1/2, cPLA2 and NF-κB in microglia suppression by cannabinoid receptor agonists and antagonists. Prostaglandins Other Lipid Mediat.

[35] Guo K, Mou X, Huang J, Xiong N, Li H (2014). Trans-caryophyllene suppresses hypoxia-induced neuroinflammatory responses by inhibiting NF-κB activation in microglia. J Mol Neurosci.

[36] Popiolek-Barczyk K, Mika J (2016). Targeting the microglial signaling pathways: new insights in the modulation of neuropathic pain. Curr Med Chem.

[37] Chu LW, Chen JY, Wu PC, Wu BN (2015). Atorvastatin prevents neuroinflammation in chronic constriction injury rats through nuclear NFκB downregulation in the dorsal root ganglion and spinal cord. ACS Chem Neurosci.

[38] Luo JG, Zhao XL, Xu WC, Zhao XJ, Wang JN, Lin XW, Sun T, Fu ZJ (2014). Activation of spinal NF-κB/p65 contributes to peripheral inflammation and hyperalgesia in rat adjuvant-induced arthritis. Arthritis Rheumatol.

[39] Wang Z, Ma W, Chabot JG, Quirion R (2010). Calcitonin gene-related peptide as a regulator of neuronal CaMKII-CREB, microglial p38-NFκB and astroglial ERK-Stat1/3 cascades mediating the development of tolerance to morphine-induced analgesia. Pain.

[40] Ma X, Jia YT, Qiu DK (2007). Inhibition of p38 mitogen-activated protein kinase attenuates experimental autoimmune hepatitis: involvement of nuclear factor kappa B. World J Gastroenterol.

[41] Gong Y, Xue B, Jiao J, Jing L, Wang X (2008). Triptolide inhibits COX-2 expression and PGE2 release by suppressing the activity of NF-kappaB and JNK in LPS-treated microglia. J Neurochem.

[42] Zhou YL, Jiang GQ, Wei J, Zhang HH, Chen W, Zhu H, Hu S, Jiang X, Xu GY (2015). Enhanced binding capability of nuclear factor-κB with demethylated P2X3 receptor gene contributes to cancer pain in rats. Pain.

[43] Degagné E, Grbic DM, Dupuis AA, Lavoie EG, Langlois C, Jain N, Weisman GA, Sévigny J, Gendron FP (2009). P2Y2 receptor transcription is increased by NF-kappa B and stimulates cyclooxygenase-2 expression and PGE2 released by intestinal epithelial cells. J Immunol.

[44] Park SW, Kim SR, Kim Y, Lee JH, Woo HJ, Yoon YK, Kim YI (2015). Chelidonium majus L. extract induces apoptosis through caspase activity via MAPK-independent NF-κB signaling in human epidermoid carcinoma A431 cells. Oncol Rep.

[45] Oita RC, Ferdinando D, Wilson S, Bunce C, Mazzatti DJ (2010). Visfatin induces oxidative stress in differentiated C2C12 myotubes in an Akt- and MAPK-independent. NFkB-dependent manner Pflugers Arch.

[46] Wang X, Chen Q, Xing D (2012). Focal adhesion kinase activates NF-κB via the ERK1/2 and p38MAPK pathways in amyloid-β25-35-induced apoptosis in PC12 cells. J Alzheimers Dis.

[47] Zhang W, Rojas M, Lilly B, Tsai NT, Lemtalsi T, Liou GI, Caldwell RW, Caldwell RB (2009). NAD(P)H oxidase-dependent regulation of CCL2 production during retinal inflammation. Invest Ophthalmol Vis Sci.

[48] Jin D, Yang JP, Hu JH, Wang LN, Zuo JL (2015). MCP-1 stimulates spinal microglia via PI3K/Akt pathway in bone cancer pain. Brain Res.

[49] Lee JY, Jhun BS, Oh YT, Lee JH, Choe W, Baik HH, Ha J, Yoon KS, Kim SS, Kang I (2006). Activation of adenosine A3 receptor suppresses lipopolysaccharide-induced TNF-alpha production through inhibition of PI 3-kinase/Akt and NF-kappaB activation in murine BV2 microglia cells. Neurosci Lett.

